# Novel Thiol Containing Hybrid Antioxidant-Nitric Oxide Donor Small Molecules for Treatment of Glaucoma

**DOI:** 10.3390/antiox10040575

**Published:** 2021-04-08

**Authors:** Charles E. Amankwa, Sudershan R. Gondi, Adnan Dibas, Courtney Weston, Arlene Funk, Tam Nguyen, Kytai T. Nguyen, Dorette Z. Ellis, Suchismita Acharya

**Affiliations:** 1 Department of Pharmacology and Neuroscience, University of North Texas Health Science Center, Fort Worth, TX 76107, USA; CharlesAmankwa@my.unthsc.edu (C.E.A.); sudershan.gondi@unthsc.edu (S.R.G.); dibasa@yahoo.com (A.D.); courtney.weston@outlook.com (C.W.); Arlene.Funk@my.unthsc.edu (A.F.); 2 North Texas Eye Research Institute, University of North Texas Health Science Center, Fort Worth, TX 76107, USA; dorette.ellis@unthsc.edu; 3 Department of Bioengineering, University of Texas at Arlington, Arlington, TX 76010, USA; tam.nguyen12@mavs.uta.edu (T.N.); knguyen@uta.edu (K.T.N.); 4 Department of Pharmaceutical Sciences, College of Pharmacy, University of North Texas Health Science Center, Fort Worth, TX 76107, USA

**Keywords:** nitric oxide, superoxide radical, hybrid small molecule, glaucoma, intra-ocular pressure, nanosuspension

## Abstract

Oxidative stress induced death and dysregulation of trabecular meshwork (TM) cells contribute to the increased intraocular pressure (IOP) in primary open angle (POAG) glaucoma patients. POAG is one of the major causes of irreversible vision loss worldwide. Nitric oxide (NO), a small gas molecule, has demonstrated IOP lowering activity in glaucoma by increasing aqueous humor outflow and relaxing TM. Glaucomatous pathology is associated with decreased antioxidant enzyme levels in ocular tissues causing increased reactive oxygen species (ROS) production that reduce the bioavailability of NO. Here, we designed, synthesized, and conducted in vitro studies of novel second-generation sulfur containing hybrid NO donor-antioxidants **SA-9** and its active metabolite **SA-10** to scavenge broad-spectrum ROS as well as provide efficient protection from *t*-butyl hydrogen peroxide (TBHP) induced oxidative stress while maintaining NO bioavailability in TM cells. To allow a better drug delivery, a slow release nanosuspension **SA-9** nanoparticles (**SA-9 NPs**) was prepared, characterized, and tested in dexamethasone induced ocular hypertensive (OHT) mice model for IOP lowering activity. A single topical eye drop of **SA-9 NPs** significantly lowered IOP (61%) at 3 h post-dose, with the effect lasting up to 72 h. This class of molecule has high potential to be useful for treatment of glaucoma.

## 1. Introduction

Primary open angle glaucoma (POAG) is among the leading causes of irreversible blindness globally. POAG is associated with compromised trabecular meshwork (TM), decreased aqueous humor outflow, increased intraocular pressure (IOP) [1], and degeneration of optic nerve head, altogether resulting in progressive vision loss. Despite damage to the TM, POAG is considered a multifactorial disease in which aging, inflammation, neurotrophic factors, genetics, and oxidative stress may play key roles in the pathophysiology [2]. However, the underlying mechanism still remains unclear. Elevated IOP is an important risk factor for the development and progression of POAG. In general, the IOP elevation in glaucoma is caused by the increased resistance to aqueous humor outflow, leading to ischemia and decreased oxygen supply to the retina, which progressively results in retinopathy. Treatment options have essentially focused on reducing IOP and increasing aqueous humor outflow or decreasing aqueous humor formation. A large variety of drug classes including alpha 2 adrenergic agonist, beta blockers, prostaglandin F2 analogues, and carbonic anhydrase inhibitors have been successfully used for IOP reduction over two decades. However, there is an unmet need for new agents to treat glaucomatous optic neuropathy that will decrease cellular dysfunction and death associated with both TM cells and retinal ganglion cells (RGCs) as well as possess IOP lowering capability. Moreover, the gap in the development of a sustained delivery system for topical ocular drug classes limits the effective control of IOP. Thus, many patients require more than one agent to achieve target IOP values with a potential challenge of non-compliance [3]. Nitric oxide (NO) is an essential biological modulator of IOP homeostasis. It is directly implicated in vasodilation via increasing cyclic guanosine monophosphate (c-GMP) in TM and ciliary epithelial cells leading to increased aqueous humor outflow, decreased episcleral venous pressure and lower IOP [4]. Vyzulta is a NO-donating prostaglandin analog currently approved by FDA for glaucoma [5]. Along with IOP lowering benefit, picomolar to nanomolar concentration of NO is desired for the beneficial physiological activities while a higher concentration is detrimental to cells and neurons. Endothelial nitric oxide synthetase (eNOS) plays a key role in the protection of the neurovascular system, and the activation of eNOS is required for neuroprotection against ischemic stroke in patients with diabetes [6]. The production of NO derived from eNOS around the nerve vessels is capable of regulating the tension between cerebral vessels and plays a positive role in improving the blood supply of the brain tissue [7]. An imbalance in the cellular redox state involving the generation of excess reactive oxygen species (ROS) and dysfunction in the antioxidant system play important roles in the pathogenesis of POAG [8]. Decrease in antioxidant enzymes including superoxide dismutase (SOD) and glutathione peroxidase levels are reported in POAG patient eyes. In an IOP-induced retinal ischemic rat model, intravitreal administration of SOD showed significant functional retina recovery when assessed using electroretinogram [9]. Due to the decrease in antioxidant enzymes, superoxides (O_2_^−^) and other ROS produced can combine with NO to form toxic peroxynitrite (ONOO^−^) radical and decrease the NO bioavailability. Therefore, it is desired to neutralize/scavenge the excess free radicals and maintain a physiological balance of NO level in ocular tissues.

**Figure 1:**
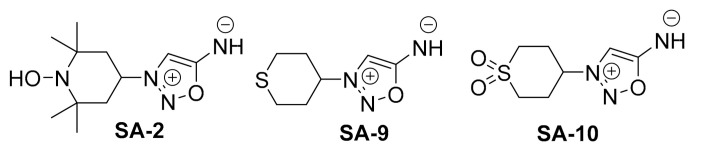
Chemical structures of the hybrid anti-oxidant, nitric oxide (NO) donors.

Previously [10], we synthesized novel bi-functional hybrid compound **SA-2** with both NO donating and SOD mimetic activity. Compound **SA-2** is neuroprotective both in ex vivo hypoxic insult of adult rat retinal explants and in the in vivo mouse optic nerve crush model [11] and lower IOP in rodent eyes [12]. Here, we hypothesize that the next generation sulfide analog **SA-9** and its active oxidative sulfoxide metabolite **SA-10** (Figure 1) will offer improved antioxidant activity and better cytoprotection to TM cells than the first generation hybrid SA-2 while maintaining NO bioavailability sufficient to provide IOP lowering activity. In the present study, we demonstrated the broad-spectrum anti-oxidant activity and NO release of **SA-9** and **SA-10** in both buffered saline and TM cell supernatants in scavenging superoxides, hypochlorous (HOCl) radicals, and produce less peroxynitrite radicals than the first-generation hybrid SA-2. We further evaluated the release profile of **SA-9** nanoparticle (NP) formulation in PBS, and conducted a biodistribution study in mouse eyes. Later, we evaluated the IOP lowering activity after a single eye drop administration of **SA-9 NPs** in dexamethasone induced ocular hypertensive mouse model to assess the IOP lowering activity and duration of action.

## 2. Methods

### 2.1. Materials and Reagents

Dexamethasone, WST-1, pyrogallol, DTNB, and all the reagents for synthesis of SIN-1, **SA-2**, **SA-9**, and **SA-10** were purchased from Sigma Aldrich. NTM-5 cells were grown from human donor eyes with no history of ocular disease or surgery and were obtained from Lions Eye Institute (Tampa, FL) or from human TM explants obtained from corneal scleral rims stored in medium (Optisol or Dexol; Chiron Ophthalmics, Irvine, CA, USA) at 4 °C. Transformed TM cells were generous gifts of Abbot F. Clark to Dorette Ellis (Alcon Laboratories, Fort Worth, TX, USA) [13] MTT reagent was purchased from Promega. Peroxynitrite assay kit was purchased from abcam. SA-2 and SIN-1 were synthesized in-house using our previously reported procedure [10].

### 2.2. Synthesis of **SA-9** and **SA-10**

Synthetic Scheme: Compounds **SA-9** and **SA-10** are prepared from commercially available amines 1 and 2) in three steps. 
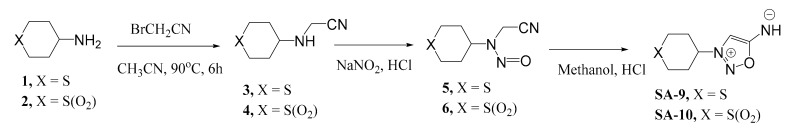


#### 2.2.1. Synthesis of Compound **SA-9**

**2-((Tetrahydro-2H-thiopyran-4-yl)amino)acetonitrile (3):** To a mixture of amine (**1**, 500 mg, 4.23 mmol, 1.0 eq) in acetonitrile (20 mL) was added successively 648 mg (4.70 mmol, 1.1 eq) of potassium carbonate and bromo acetonitrile (560 mg, 3.68 mmol, 1.1 eq). The mixture was refluxed for 6 h. The solids were filtered and washed with 9:1 ratio of chloroform:methanol (90 mL:10 mL). The filtrate was concentrated to provide 600 mg residual oil, which was dissolved in 10 mL of methanol, and triturated with diethyl (100 mL). After 30 min of stirring, the yellow solid was filtered, and dried under vacuum to obtain 500 mg of product **3** in 74.9% yield. ^1^H NMR (300 MHz, DMSO-d_6_): δ10.49 (br-s, 1H, NH), 4.35 (s, 2H), 3.18–3.11 (t, 1H, J = 10.5 Hz), 2.72–2.62 (m, 4H), 2.36–2.33 (m, 2H), 1.72–1.69 (m, 2H). ^13^C-NMR (75 MHz, DMSO-d_6_): δ 114.70 (-CN), 56.14(-C-NH-), 30.93(-CH_2_CN), 30.07(C-S-), 26.48(C-CH_2_S-). TOF-Mass: C_7_H_12_NS (M + H): 157.25.

**(3-(tetrahydro-2H-thiopyran-4-yl)-1,2,3-oxadiazol-3-ium-5-yl)amide hydrochloride (SA-9):** Compound **3** (0.8 mmol, 130 mg) was dissolved in 10 mL of 6N HCl at 0 °C and 250 mg (5.0 eq) of NaNO_2_ was added to it, and the reaction mixture was stirred at 0 °C for 1 h. The inorganic solids were filtered. The reaction mixture was extracted with EtOAc (25 mL × 2) to remove the organic impurities. The colorless aqueous layer on concentration provided sticky solids which was dissolved in methanol (5 mL) and triturated with diethyl ether (40 mL) to precipitate the solid out. The solid was filtered, washed with diethyl ether (10 mL), and dried under vacuum to obtain 130 mg of **SA-9** white solid in 71% yield. ^1^H NMR (300 MHz, D_2_O): δ 7.61 (s, 1H), 4.89–4.78 (m, 1H), 2.87–2.71 (m, 4H), 2.50–2.46 (m, 2H), 2.20–2.07 (m, 2H). ^13^C-NMR (75 MHz, D_2_O): δ 169.54 (O-**C**-NH), 101.40 (N-**C**=), 61.84 ((-**C**-N=), 43.07 **C**-S-), 22.61 (**C**-CH_2_S-). FT-IR (neat) cm^−1^: 2910, 1667, 1471, 1179, 1027, 944. TOF-Mass: C_7_H_11_N_3_OS (M + H): 186.0673.

#### 2.2.2. Synthesis of Compound **SA-10**

**2-((1,1-dioxidotetrahydro-2H-thiopyran-4-yl)amino)acetonitrile (4):** The intermediate **4** was prepared from amine **2** following the same synthetic procedure as described for **3** in 79.2% yield. ^1^H NMR (300 MHz, DMSO-d_6_): δ 4.39 (s, 2H), 3.51–3.43 (m, 1H), 3.34–3.20 (m,4H), 2.41–2.38 (m, 2H), 2.17–2.04 (m, 2H). ^13^C-NMR (75 MHz, DMSO-d_6_): δ 114.62 (-**C**N), 52.81(-**C**-NH-), 48.06(**C**-SO_2_-), 32.02(-**C**H_2_CN), 26.48(**C**-CH_2_SO_2_). TOF-Mass: C_7_H_12_N_2_O_2_S (M + H): 189.0600.

**Method A: N-(cyanomethyl)-N-(1,1-dioxidotetrahydro-2H-thiopyran-4-yl)nitrous amide (6):** To a pre-cooled solution of 1.6 mmol (300 mg) nitrile **4** in acidic H_2_O (10 mL containing 1.7 mL of 1N HCl) at 0 °C, 120 mg of NaNO_2_ was added and the reaction mixture was stirred at 0 °C for 1 h to form a white precipitate. The pale white solid was filtered, washed with n-hexane, and dried under vacuum to get 220 mg of pale white solid in 64% yield as the nitroso intermediate **6**. ^1^H NMR (300 MHz, DMSO-d_6_): δ 4.97–4.83 (m, 1H), 4.72 (s, 2H), 3.50–3.40 (m, 2H), 3.38–3.20 (m, 2H), 2.51–2.30 (m, 4H). ^13^C-NMR (75 MHz, DMSO-d_6_): δ 114.40 (-**C**N), 59.31(-**C**-NH-), 49.44 (**C**-SO_2_-), 30.82 (-**C**H_2_CN), 28.88 (**C**-CH_2_SO_2_-). TOF-Mass: C_7_H_11_N_3_O_3_S (M + H): 218.0600.

**(3-(1,1-dioxidotetrahydro-2H-thiopyran-4-yl)-1,2,3-oxadiazol-3-ium-5-yl) amide hydrochloride (SA-10):** The nitroso compound **6** (200 mg, 0.92 mmol) was suspended in methanol (5 mL) followed by the addition of 3 mL of 1 M methanolic HCl. The reaction mixture was stirred overnight at room temperature to form white salt, which was filtered, washed with diethyl ether (20 mL), and dried under vacuum to obtain 175 mg of **SA-10** as white solid in 75% yield. ^1^H NMR (300 MHz, D_2_O): δ 7.75 (s, 1H), 5.26–5.17 (m, 1H), 3.51–3.35 (m, 4H), 2.73–2.70 (m, 4H). ^13^C-NMR (75 MHz, D_2_O): δ 169.56 (O-**C**-NH), 102.00 (N-**C**=), 60.41 ((-**C**-N=), 48.00 **C**-SO_2_-), 28.53 (**C**-CH_2_SO_2_). FT-IR (neat) cm^−1^: 2993, 1705, 1509, 1338, 1122, 957. TOF-Mass: C_7_H_11_N_3_O_3_S (M + H): 218.0600.

**Method-B:** Compound **4** (1.6 mmol, 300 mg) was dissolved the in 20 mL of 6N HCl at 0 °C, and 250 mg (5.0 eq) of NaNO_2_ was added to it, and the reaction mixture was stirred at 0 °C for 1 h. The inorganic solid was filtered, then extracted with EtOAc (2 × 25 mL) to remove the organic impurities. The aqueous layer on concentration provided a sticky solid which was dissolved in methanol (10 mL) and triturated with diethyl ether (75 mL) to precipitate. The precipitate was filtered, washed with diethyl ether (25 mL), and dried under vacuum to obtained 380 mg of white solid **SA-10** in 93% yield.

### 2.3. Superoxide Anion Radical Scavenging Assay

The ROS scavenging ratio was determined using a chemical assay where WST-1 was used as superoxide scavenger produced by pyrogallol following a reported protocol [14]. Briefly, the superoxide anion radical scavenging activity was determined spectrophotometrically by measuring the formation of WST-1 formazan at 450 nm. WST-1 (40 µL, 50 µM) was added to the compounds (or corresponding solution) in a final volume of 160 µL in 50 mM ammonium hydrogen carbonate buffer, 0.5 mM EDTA, pH 9.3. Reaction was launched by adding 40 µL 1 mM pyrogallol. The operation was based on the 96-well microplate, and measurement was performed at different time points under 37 °C by Cytation5 Microplate Reader. Baicalein (1 mM) was used as positive control. Compounds SA-2, SA-9, and SA-10 were used in concentrations ranging from 1 µM, 10 µM, 100 µM, and 1000 µM. The superoxide anion radical scavenging ratio % was calculated according to the following formula:

Scavenging ratio (%) = A_0_ − (A_1_ − A_2_)/A_0_ × 100%

### 2.4. Hypochlorous Acid (HOCl) Radical Scavenging Assay

This assay used the redox reaction between 5-thio-2-nitrobenzoic acid (TNB) and HOCl to form dithio-TNB. Using the method described previously [15], we assessed the % TNB remaining with or without treatment with SA compounds. Compounds SA-2, SA-9, and SA-10 were used in concentrations ranging from 0.1 µM, 1 µM, 10 µM, 100 µM, and 1000 µM. Lipoic acid (1 mM) was used as positive control.

### 2.5. Measurement of Total Nitrite Release by Griess Assay

The total nitrite released from the compounds in buffer and in cell supernatant were measured by Griess assay as reported by us previously [10]. In brief, compounds (**SA-2**, **SA-9**, and **SA-10**) in buffer at different concentrations (1 mM, 500 µM, 250 µM 125 µM, 62.5 µM, 31.25 µM, and 15.625 µM) or supernatant collected from Human transformed trabecular meshwork (NTM-5) cells were added to a 96-well plate. 1% Sulphanilamide in 5% phosphoric acid and 0.1% N-1-napthylethylenediamine dihydrochloride (NED) were added to experimental samples sequentially at room temperature and away from light. SIN-1 was used as reference standard. UV Absorbances (Cytation 5) were measured at specific timepoints (0, 5 min, 10 min, 15 min, 30 min, 24 h, and 28 h) at 520 nm.

### 2.6. In Vitro Peroxynitrite Measurement Assay

Human transformed trabecular meshwork (NTM-5) cells (20,000 cells/well/90 µL) were seeded in 96-well plates (Griener bio-one black plate) and stained with 10 µL of 10X Peroxynitrite Sensor Green solution for 1 h. The stained NTM-5 cells were treated with different concentrations of SA compounds (1, 10, 100, and 1000 µM) away from light at 37 °C and 5% CO_2_ saturation. SIN-1 was used as a positive control. Detection of peroxynitrite was done using Peroxynitrite Assay Kit (abcam-ab233468-cell based). Fluorescence signals were measured using a microplate reader (Cytation5) at Ex/Em = 485/530 nm at time intervals (15 min, 30 min, 1 h, 2 h, and 3 h). The experiments were done in duplicates.

### 2.7. In Vitro Cell Viability Assay

NTM-5 cells (6000 cells/well) were seeded in 96-well plates to confluency. The cells were starved for 48 h with no serum media and exposed to different concentrations of SA compounds (0.1, 1, 10, 100, and 1000 µM). After 24 h of incubation at 37 °C, the cell supernatant was stored at −80 °C for Griess assay as described above for determination of total nitrite concentrations. The cell viability and proliferation were measured using MTT assays (CellTiter 96^®^ AQueous One Solution Cell Proliferation Assay, Promega Madison, WI, USA) following manufacturer instruction. The experiments were repeated twice with four technical replicates.

### 2.8. In Vitro Cytoprotection Assay

NTM-5 cells were starved for 48 h with no serum media and then treated with *t*-butyl hydrogen peroxide (TBHP, 350 µM) for 30 min followed by co-treatment with SA compounds at different concentrations (1µM, 10 µM, 100 µM, and 1000 µM). **SA-9 NPs** or blank NPs were used in 1% *w/v* formulated in PBS. After 24 h of treatment, cell proliferation was measured using MTT assay. All the experiments are done in duplicate with four experimental replicates.

### 2.9. Fabrication of **SA-9NPs**

The fabrication and characterization of **SA-9 NPs** was conducted similar to the protocol previously described by us [16]. In brief, 10 mg of **SA-9** was dissolved 100 µL DMSO and then transferred to 3 mL of chloroform containing 90 mg of Polylactic-co-glycolic acid (PLGA) to form an oil phase. This solution was then added dropwise into 20 mL of 5% PVA solution (water phase) followed by sonication at 25 W for 1 min to form the **SA-9 NPs**. The emulsion was stirred overnight to completely evaporate the organic solvent. Next, the NPs were pelleted by ultracentrifugation at 20,000 rpm for 20 min followed by washing twice with DI water. Finally, the NP pellet was dissolved in DI water and lyophilized to obtain a powder form.

### 2.10. Characterization of **SA-9NPs**

Size and zeta potential of **SA-9 NPs** were determined by Brookhaven dynamic light scattering device (DLS, Brookhaven Instruments Co. New York, USA). Nanoparticle morphology was observed via Hitachi transmission electron microscopy (TEM, H-9500). **SA-9 NPs** stability in saline and simulated body fluid (SBF) was confirmed by checking size changes at different time points over 48 h. Drug loading efficiency and drug release profile were quantified by measuring **SA-9** absorbance (240 nm from 200–1000 spectrum) via Tecan spectrophotometry. For **SA-9** loading efficiency, free **SA-9** was diluted in a similar solvent to the sample solvent at different concentration to make the standard curve. The supernatant of **SA-9 NPs** fabrication was used to measure the amount of unloaded **SA-9**. Then, **SA-9** loading efficiency was calculated by the following Equation (1):

(1) $$SA\text{-}9\;loading\;efficiency\;\left(\%\right)\;=\;\frac{\left(Initial\;SA\text{-}9\right)-\left(Unloaded\;SA\text{-}9\right)}{\left(Inital\;SA\text{-}9\right)}\times 100\%$$

1 mL of **SA-9 NPs** suspension (5 mg/mL) in PBS 1X (pH_7.40_) was placed in a dialysis bag with MWCO 3.5–5 kDa (Spectrum, Catalog 131192), submerged in 20 mL PBS 1X pH 7.40 (so-called dialysate) and shaken at 37 °C over a time range. At each time point, 1 mL of dialysate solution was pooled and replaced with the same volume of fresh PBS. Each sampling solution was then read for its absorbance value and the amount of released **SA-9** was quantified against the **SA-9** standard curve. Consequently, a cumulative release profile of **SA-9** over time was plotted.

### 2.11. Animals

Animal studies were performed in accordance with the Association for Research in Vision and Ophthalmology (ARVO) resolution for the Use of Animals in Ophthalmic and Vision Research and approved by the University of North Texas Health Science Center (UNTHSC) Institutional Animal Care and Use Committee (IACUC-2019-0036 and IACUC-2017-0024). C57BL/6J mice (female, 10–12 weeks) were used in the study (*n* = 5–6 animals/group were purchased from the Jackson Laboratory, Bar Harbor, ME, USA). Mice were maintained on a 12-h light/12-h dark cycle (lights on at 0600 h) with food and water available ad libitum.

### 2.12. Formulation and Storage

Either free drugs **SA-9** or **SA-9NPs** (weight/volume, *w/v*) were reconstituted in sterile phosphate buffer saline (PBS) 7.40 (1×) and vortexed to obtain a solution or a milky white nanosuspension, respectively. All the formulations were freshly prepared before dosing and the left overs were discarded.

### 2.13. Ocular Biodistribution after **SA-9** and **SA-9NPs** Eye Drop

Using an in-house developed HPLC/MS method, the concentration of compound **SA-9** was measured in the lens, retinal, choroidal, scleral, and optic nerve (ON) tissues 1 h and 24 h post dosing (30 µL topical ocular eye drop) of either 2% of **SA-9** or 2% and 4% *w/v* of **SA-9 NPs** in mouse eyes (*n* = 12) following a modified protocol for mouse eyes [11,17].

### 2.14. IOP Lowering Activity of **SA-9 NPs** in Dexamethasone Induced Ocular Hypertensive (OHT) Mouse Eyes

Mice (C57BL/6J, 12 weeks, *n* = 3–5) were anesthetized and the periocular injection of dexamethasone acetate (DEX-Ac, 10 mg/mL) or vehicle (20 μL) was administered immediately under the isoflurane over the course of 10 to 15 s. The procedure was performed in both eyes of each mice after it was numbed with proparacaine as reported previously [18]. Mice were treated with DEX-Ac or vehicle once per week for four weeks to achieve the desired IOP elevation. Baseline IOP was measured using tonometer. Compound **SA-9 NPs** suspension formulated in sterile PBS was instilled as a single eye drop (5 µL), and IOP was measured at different time points using TonoLab rebound tonometer (TonoLab; Tiolat Oy; Helsinki, Finland) as described previously [19] at various time points: 0, 3, 6, 24, 48, and 72 h post-dosing. The entire dosing schedule was repeated 3 times in each mouse eye. IOP plots were generated from IOP values obtained from the treated eyes.

## 3. Results

### 3.1. Anti-Oxidant Activity of SA Compounds

**Figure 2:**
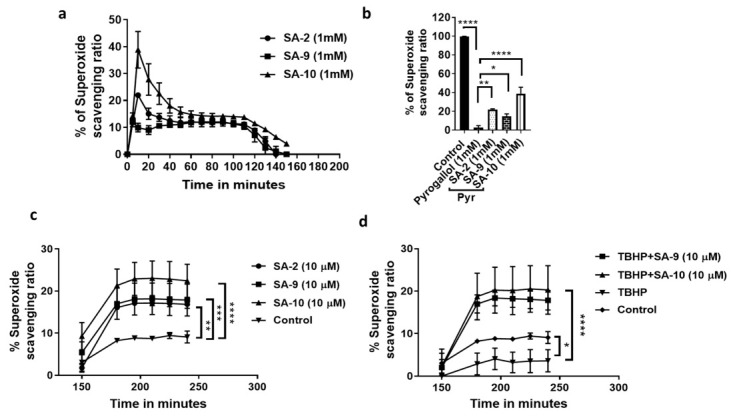
Superoxide scavenging ability of SA compounds in buffer and cells. (**a**) The % of superoxide scavenging response from 1 mM concentration of **SA-2**, **SA-9**, and **SA-10** in pyrogallol (Pyr, 1 mM) induced superoxide induction assay at different time points in buffer. The AUC_0–2.5 h_ for **SA-2, SA-9**, and **SA-10** were 1592 ± 37.87, 1355 ± 42.62, and 2350 ± 69.26, respectively. (**b**) Comparison of the % ROS scavenging activity of **SA-2, SA-9**, and **SA-10** with control after 15 min of treatment of pyrogallol. (**c**) The superoxide scavenging ratio (%) after 18 h treatment of 10 µM concentration of **SA-2, SA-9**, and **SA-10** to NTM-5 cell supernatant using pyrogallol induced superoxide induction assay at different time points. (**d**) The superoxide scavenging ratio (%) of 10 µM concentration of **SA-2, SA-9**, and **SA-10** in NTM-5 cell supernatant. The cells were previously treated with 5.5 mM of TBHP for 30 min followed by SA compounds and incubated for 18 h. Control is untreated cells. Three technical replicates were used for each experiment and all experiments were repeated 2 times. * *p* < 0.05, ** *p* < 0.005, *** *p* < 0.001, **** *p* < 0.0001, One-way ANOVA for (**b**), and two-way ANOVA for (**c**,**d**).

The goal of the synthesis and in vitro evaluation of the sulfur containing hybrid compounds was to establish if such chemical modification will improve the scavenging ability of the compounds for broad-spectrum ROS and not only be limited to superoxide radicals. Earlier, we had reported that compound **SA-2** with a SOD mimetic functionality was successful in reducing the superoxide load and increasing the level of SOD enzyme in retina [11]. Here, by using two different assays, we evaluated the structure activity relationships of the newly synthesized sulfide analog **SA-9** and its oxidized sulfone metabolite **SA-10**. Utilizing a reported pyrogallol induced superoxide anion radical production assay [14], we screened the SA compounds at different concentrations (1 µM, 10 µM, 100 µM, and 1000 µM) in buffer at different time points (0–3 h) (Figure 2a). All three compounds (**SA-2**, **SA-9**, and **SA-10**) were able to scavenge superoxide radicals (16–40%) as shown in the dose response result (Figure S1a). At 1 mM concentration, **SA-10** was found to be 2.5-fold more potent than **SA-2** (Figure 2b). In NTM-5 cell supernatant, we found that the superoxide scavenging ability of **SA-10** (the oxidative metabolite of **SA-9**) is highest, followed by **SA-9** and **SA-2**. A10-fold lower (10 µM) concentration in cells was enough to show the protective activity than in buffer and was significantly higher than control cells (Figure 2c). The dose response results for all three compounds were presented in Figure S1b–e where a range of concentrations (0.1 µM, 1 µM, 10 µM, 100 µM, and 1000 µM) were evaluated. Next, when the cells were insulted with an oxidant TBHP to mimic glaucoma-like oxidative insult, the superoxide scavenging ability of the TBHP treated TM cells significantly went down (10% to 2.5%), however, when the cell were treated with 10 µM of either **SA-9** or **SA-10** for 18 h, the decrease in superoxide scavenging ability was significantly prevented (~10 fold, 20–25% vs. 2.5%) as shown in Figure 2d.

**Figure 3:**
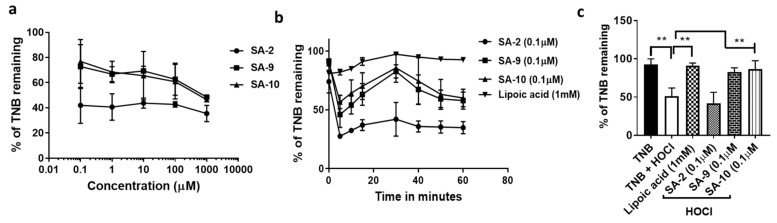
The HOCl scavenging activity of SA compounds in buffer solution. (**a**) Addition of oxidant HOCl to TNB decreased the % of TNB to ~50% as measured by absorbance at 520 nm. Addition of compounds at different concentrations (0.1 µM, 1 µM, 10 µM, 100 µM, and 1000 µM) of SA compounds **SA-2**, **SA-9**, and **SA-10** rescued the decrease in fluorescence of TNB measured at 30 min, which is proportional to the HOCl scavenging activity. (**b**) Addition of compounds at 0.1 µM rescued the decrease in fluorescence of TNB measured at different time points, which is proportional to the HOCl scavenging activity. Lipoic acid (1 mM) was used as positive control. (**c**) The % of TNB remaining after 30 min of addition of HOCl to the wells containing lipoic acid, **SA-2, SA-9**, and **SA-10**. *n* = 4, ** *p* < 0.001. One-way ANOVA, Graph Pad Prism.

In the next study, we assessed the scavenging ability of another highly cytotoxic free radical hypochlorous acid (HOCl) that is also known to decrease the NO bioavailability. Activated neutrophils release oxidants like O_2_.^−^, H_2_O_2_, and OH radicals. The enzyme myeloperoxidase uses H_2_O_2_ to oxidize chloride ions into HOCl, which combine with NO_2_^−^ and facilitate the protein nitrotyrosylation leading to inflammation and tissue injury. HOCl imparts a defect in endothelial NO production due to a superoxide-dependent reduction in endothelial nitric oxide synthase dimer stability and contribute to the impairment of NO bioactivity and bioavailability [20]. Earlier reports demonstrated that, presence of sulfide and sulfone improves the HOCl scavenging activity in a compound and was well exemplified by the superior HOCl scavenging activity of the sulfide and sulfone metabolites of Sulindac [15]. As expected, both **SA-9** and the oxidative metabolite **SA-10** were equally efficacious in scavenging HOCl at different doses 0.1 µM–1000 µM in buffer with the effect lasting >1 h (Figure 3a,b) and are 10,000 fold more potent (0.1 µM vs. 1 mM) than the positive control lipoic acid (Figure 3c).

### 3.2. Compounds **SA-9** and **SA-10** Release Nitric Oxide in Cells but Not in Buffer Formulation

**Figure 4:**
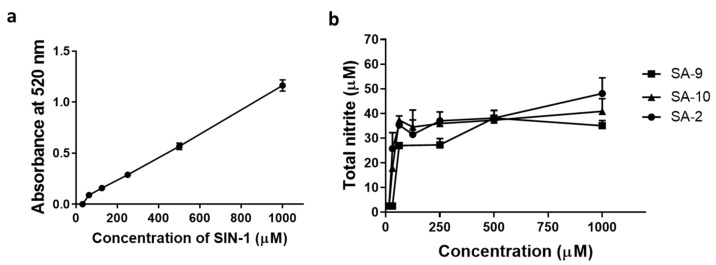
(**a**) Different concentrations of SIN-1 (31.25 µM, 62.5 µM, 125 µM, 250 µM, 500 µM, and 1000 µM), a known NO donor was used as reference standard. (**b**) Total nitrite release from NTM-5 cells after 28 h of treatment with different concentrations of **SA-2, SA-9**, and **SA-10** as determined using Griess assay. *n* = 3.

One of the important goals in this hybrid compound design was to identify compounds with good aqueous stability and half-life at a pH of 6.5–7.5 to aid in topical ocular drug formulation. Earlier, we reported that the hybrid sydnonimine-Tempo compound **SA-2** possesses a short half-life in PBS_7.4_ (<1 h) and time and dose dependently release NO as detected by using Griess assay [10]. Here, we did not observe any NO release from both **SA-9** and **SA-10** in buffer even after 3 days indicating improved aqueous stability than **SA-2**. As expected, in NTM-5 cell supernatants we were able to detect the NO released from all three compounds **SA-2**, **SA-9**, and **SA-10** dose dependently after incubating for 28 h at 37 °C (Figure 4b). SIN-1, a known NO donor was used as standard control (Figure 4a).

### 3.3. Compounds **SA-9** and **SA-10** Dose Dependently Protect TM Cells from Oxidative Stress Induced Cell Death

**Figure 5:**
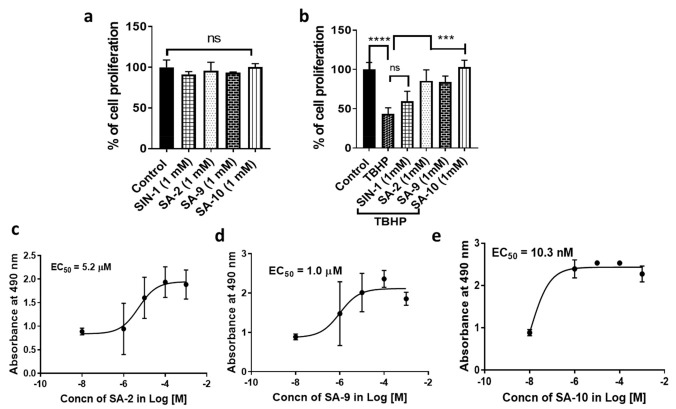
Effect of SA compounds in NTM-5 cell proliferation. (**a**) The % of cell proliferation measured by MTT assay represented cell viability after treatment of SA compounds to NTM-5 cells for 18 h of treatment. (**b**) Treatment with TBHP (350 µM) significantly decreased the NTM-5 cell proliferation. Treatment with SA compounds rescued the cell from death. (**c**–**e**) Compounds **SA-2, SA-9**, and **SA-10** dose-dependently increased the number of viable NTM-5 cells that was decreased significantly in the presence of *tert*-butyl hydroperoxide (TBHP, 350 µM). Experiments were repeated three times with *n* = 4 technical replicates. Values are expressed in mean ± standard error of the mean (SEM). EC_50_ values were calculated using GraphPad Prism. *** *p* < 0.001, **** *p* < 0.0001. One-way ANOVA, Graph Pad Prism.

Here, we used a transformed trabecular meshwork cell line (NTM-5), for compound screening that provided reproducible results. First, we determined the half-maximal effective concentration (EC_50_) dose of TBHP in NTM-5 cells via dose response study and found that to be 350 µM. In the next study, a dose response study was conducted to determine the cytotoxicity of **SA-2, SA-9**, and **SA-10** in NTM5 cells after 24 h of treatment at different concentrations (1, 10, 100, and 1000 µM). We found no cytotoxicity even at a high dose of 1000 µM for all three compounds ( Figure 5a). TM cell viability was significantly decreased when treated with 350 µM of TBHP (Figure 5b). Treatment with compounds **SA-2**, **SA-9**, and **SA-10** significantly and dose-dependently increased the cell viability, and their EC_50_ were calculated to be 5.2 µM, 1.0 µM, and 10.3 nM, respectively (Figure 5c–e). The sulfone compound **SA-10** was found to be 10-fold more potent than the sulfide compound **SA-9**. This result provides ~1000-fold therapeutic index for **SA-9** (1.0 µM vs. 1000 µM) and ~10,000-fold therapeutic index for **SA-10** (10.3 nM vs. 1000 µM) over the safety dose used in the cell cytotoxicity study using NTM-5 cells. Compound **SA-2** was found to be less potent in this cell line (EC_50_ = 5.2 µM).

### 3.4. Effect of SA Compounds in Peroxinitrite Radical Formation in NTM5 Cells

**Figure 6:**
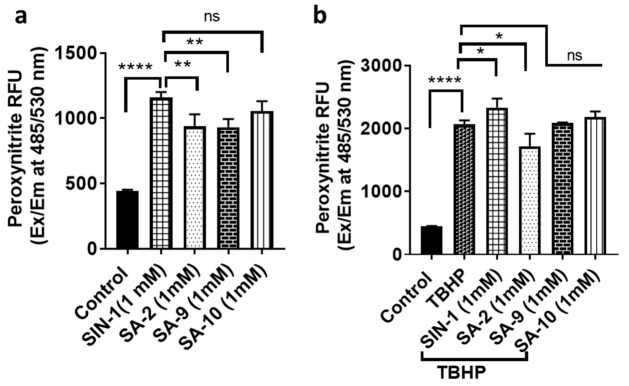
Quantitative measurement of ONOO^−^ radical generation in NTM-5 cells. (**a**) NTM-5 cells were labelled with peroxynitrite green sensor followed by treatment with 1 mM of SIN-1, **SA-2**, **SA-9**, and **SA-10**. Changes in green fluorescence signal corresponded to level of ONOO^−^ radicals as measured at Ex/Em of 485/530 nm. (**b**) NTM-5 cells labelled with peroxynitrite green sensor followed by treatment with TBHP (350 µM) and 1 mM of SIN-1, **SA-2, SA-9**, and **SA-10**.. Changes in green fluorescence signal corresponded to the level of ONOO^−^ radicals as measured at Ex/Em of 485/530 nm. *n* = 3, * *p* < 0.05, ** *p* < 0.001, **** *p* < 0.0001. One-way ANOVA, Graph Pad Prism.

We conducted a fluorometric assay to detect peroxynitrite radicals (ONOO^−^) in NTM-5 cells. SIN-1 is a known ONOO^−^ radical generating compound. SIN-1 possesses the sydnonimine NO donor group, which at biological pH generates one molecule of NO and one molecule of O_2_^−^ radical that self-associate to form ONOO^−^ radicals. In NTM-5 cell experiment, addition of SIN-1 [21] produced significantly higher level of ONOO^−^ than that from **SA-2** or **SA-9** (Figure 6a). Additionally, when the cells were further stressed with TBHP (350 µM) followed by addition of SIN-1 and SA compounds at two concentrations (0.1 µM and 1 mM), while **SA-2** treatment produced less ONOO^−^ radicals, SIN-1 significantly increased the radical formation (Figure 6b). We observed that the positive control SIN-1 produced similar amount of ONOO^−^ radical both at the low and high doses (Figure S2) where **SA-9** produces significantly less amount of radicals at both 0.1 µM and 1 mM doses. This observation proved our hypothesis that, though SIN-1, **SA-2, SA-9**, and **SA-10** all compounds possess the sydnonimine functionality as NO donor fragment and anticipate to produce ONOO^−^ radicals in biological pH, however, the antioxidant moieties present in **SA-2** and **SA-9** scavenged the superoxide radicals and thus limited the ONOO^−^ radical formation in cells. Compound **SA-10** was not as efficacious as **SA-9** in preventing ONOO^−^ radicals (Figure 6a) and the possible reason we can think of is that, as the sulfur present in **SA-10** is already oxidized to sulfone and no longer available to scavenge superoxide radicals like the sulfide analog **SA-9**.

### 3.5. Bioavailability of **SA-9** and **SA-9-NPs** on Mouse Eyes

**Figure 7:**
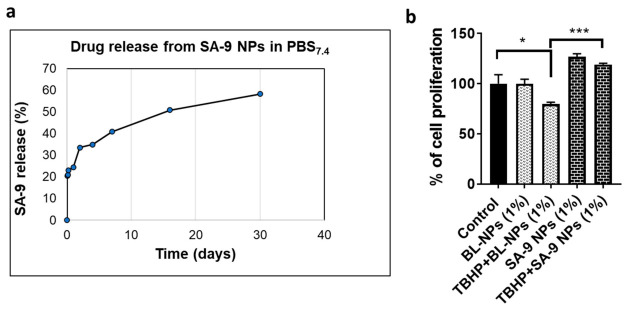
Drug release from nanoparticle in PBS and NTM-5 cells. (**a**) Drug release study of **SA-9** from its Polylactic-co-glycolic acid (PLGA) nano-encapsulated nanosuspension **SA-9 NPs** in saline at 37 °C conducted over 30 days. The UV absorbance readings are taken in triplicates at 230 nm. (**b**) Blank nanoparticles (BL-NPs) did not show any cytotoxicity to the NTM-5 cells after 18 h of treatment. Treatment with TBHP (350 µM) significantly decreased the NTM-5 cell proliferation after 18 h and co-treatment with **SA-9 NPs** (1%) rescued the cells from death. *n* = 3, * *p* < 0.05, *** *p* < 0.001. One-way ANOVA, Graph Pad Prism.

Based on the ROS scavenging activity and potent cytoprotective profile in NTM-5 cells, the sufide compound **SA-9** was selected for the formulation optimization desired for the in vivo studies. We previously found that, the hybrid molecule **SA-2** quickly hydrolyzes in physiological, aqueous environments, and an effective sustained release of **SA-2** could be achieved by packaging it in a Polylactic-co-glycolic acid (PLGA) nanoparticle carrier [16]. To ensure that **SA-9** is effective over a long period of time in vivo, we decided to encapsulate the **SA-9** in PLGA nanoparticles (**SA-9 NPs**). We determined that **SA-9 NPs** have a drug release profile with initial burst release of 22% of **SA-9** within 12 h followed by a sustained release of 35–60% over 30 days (Figure 7) in PBS at pH 7.4. The size of the nanoparticles **SA-9 NPs** was determined as 200–300 nm and UV absorbance for **SA-9** was at 230 nm.

We then tested the blank nanoparticles (BL-NPs) and **SA-9** NPs in NTM-5 cells to assess the cytotoxicity and cell viability using MTT cell proliferation assay. At 1% dose, BL-NPs did not show any cytotoxicity. We also confirmed that **SA-9** is released from SA-9 NPs (1%) and provided cytoprotection and improved cell proliferation to TBHP induced cell death in the NTM-5 cells (Figure 7b).

**Figure 8:**
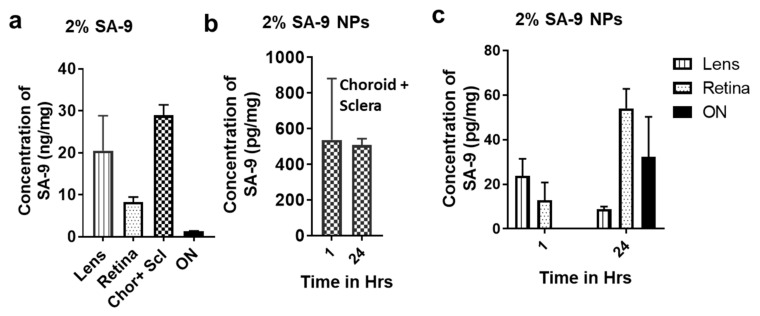
(**a**) Concentration of **SA-9** in lens, retina, choroid + sclera, and optic nerves (ON) after 1 h of single eyedrop administration of 2% **SA-9** in PBS to mice eyes. (**b**) Concentration of **SA-9** after 1 h and 24 h time-points in combined choroid and sclera tissues after single eye drop administration of 2% **SA-9 NPs** nanosuspension in PBS to mice eyes. (**c**) Concentration of **SA-9** in lens, retina, and ON at 1 and 24 h after single eye drop administration of 2% **SA-9 NPs** nanosuspension in PBS to mice eyes. No **SA-9** was detected after 1 h in ON. Blank nanoparticle was dosed to control mice and no drug was detected there. N = 12 eyes.

Based on the drug release profile, we have proceeded to conduct the bio-distribution study in mice eyes following single topical eye drop administration to each eye. Both eyes were used in each group. Four samples are combined to get 1 final sample totaling 3 samples from 12 eyes for lens, retina, choroid + sclera, and optic nerves (ON). The data is presented in Figure 8. After 1 h of instillation of a single eye drop of 2% of **SA-9** solution (Figure 8a), we observed a significant quantity (in ng/mg) of **SA-9** in lens (20.5 ± 8.3 ng/mg), retina (8.25 ± 10.2 ng/mg), choroid + sclera (28.9 ± 2.5 ng/mg), and in ON (1.30 ± 0.09 ng/mg) indicating the compound is permeable to cornea and lens as well as reaching to the back of the eye most likely via passive diffusion through sclera. The drug concentration quantified from solution formulation of **SA-9** (2% *w/v*, 90 mM) and nanosuspension **SA-9 NPs** (2% *w/v*, equivalent to 125 µM of free **SA-9**) have a linear correlation. Note that, free drug was dosed at 700-fold higher concentration than what is loaded in 2% **SA-9NPs**. As expected, we found the 2% **SA-9 NPs** provided 860-fold less concentration of **SA-9** in lens (23.81 ± 7.64 pg/mg), and 640-fold less concentration of **SA-9** in retina (Figure 8b,c). However, the combined sclera + choroid tissue **SA-9** concentration was only 54-fold less, indicating faster scleral diffusion of the nanoparticle formulation after eye drop administration. We did not see any measurable quantity of **SA-9** from 2% **SA-9 NPs** in the ON at 1 h time point, however, at 24 h time point, a pg/mg quantity of **SA-9** was detected in ON tissue (32.26 ± 18.03 pg/mg). Therefore, we concluded that both the solution and PLGA encapsulated nanosuspension formulations are suitable to deliver **SA-9** to both the front and back of the eyes after topical eye drop dosing. The small molecular weight and relatively hydrophobic nature of **SA-9** may facilitate the diffusion through cornea and sclera better and being encapsulated in PLGA nanoparticle might further enhance the scleral delivery. At 24 h time-point, more drug is found in the retina from **SA-9 NPs** and there is a linear correlation between 1 h and 24 h time points (Figure 8c). Detection of drug concentration in ON was a serendipitous finding that needs further investigation to determine if **SA-9** can be delivered further to brain and be neuroprotective there too.

Additionally, we have conducted visual observation of the eyes to understand if any toxicity occurred at the above doses. No such adverse effect was observed.

### 3.6. **SA-9NPs** Eye Drop Reduced IOP in OHT Mouse Eyes and Provided Longer Duration of Action

**Figure 9:**
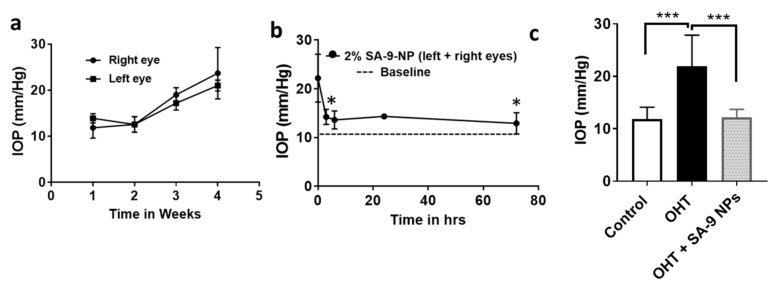
(**a**) IOP of both left and right eyes of the mice were elevated after periocular injection of DEX-Ac (10 mg/mL) once every week for 4 weeks. (**b**) IOP lowering activity of 2% **SA-9 NPs** in both OHT eyes of C57Bl/6J mouse. Treatment with 2% **SA-9 NPs** eye drop after 4 weeks of Dex-Ac injection significantly decreased the IOP in both eyes and bring to basal level. The effect lasted up to 72 h. (**c**) There is significant increase in IOP after periocular injection of DEX-Ac once a week for 4 weeks in mouse eyes, here referred as OHT group. Treatment with **SA-9 NPs** significantly decreased (−61%) the IOP at 3 h post dose. * *p* < 0.05, *** *p* < 0.005, respectively for 3 h vs. OHT groups. *n* = 6–8 eyes.

Dexamethasone is used for anti-inflammatory therapy, however, it possesses serious side effects such as elevation of IOP in humans. Increased extracellular matrix (ECM) accumulation and endoplasmic reticulum (ER) stress in the trabecular meshwork (TM) is associated with Dex-induced IOP elevation. ER stress leads to more ROS production and cell death. Zode et al. [22] reported that, the transcriptional factor C/EBP homologous protein (CHOP), a marker for chronic endoplasmic reticulum (ER) stress, is upregulated in the anterior segment tissues, and CHOP deletion reduced ER stress in these tissues and prevented dexamethasone-induced ocular hypertension. Additionally, Kasetti et al. have also reported that [23] activating transcription factor 4 (ATF4)- C/EBP homologous protein (CHOP)– growth arrest and DNA damage-inducible protein (GADD34) signaling pathway is induced in the glaucomatous TM, and that induction of these proteins is associated with IOP elevation in dexamethasone-acetate (Dex-Ac) induced OHT mice and ROS production in glaucomatous human TM cells. ATF4 levels are found to be elevated in the TM tissues of 5-week Dex-treated mice [23], indicating that this model may appropriately recapitulate the glaucoma pathology due to ROS elevation, and **SA-9** may able to scavenge them. Using a published protocol developed by Patel et al. [18], we evaluated **SA-9 NPs** eye drop in a Dex-Ac induced OHT mouse eye model for its ability to lower IOP. Dexamethasone injection once every week for 4 weeks elevated the IOP in both mouse eyes (21.9 ± 5.92 mm/Hg) compared to baseline (11.85 ± 2.27 mm/Hg) as shown in Figure 9a. A single topical eye drops of 2% **SA-9 NPs** to both eyes of the mouse that have elevated IOP, significantly lowered IOP (12.22 ± 1.48 mm/Hg, −61%) at 3 h post-dose with the effect lasting till 72 h (Figure 9b). We have not measured the IOP beyond 72 h.

## 4. Discussion

Design and synthesis of a slow-release nitric oxide (NO) donor with broad-spectrum ROS scavenging activity is anticipated to solve several fundamental issues. The multifactorial pathology of glaucoma includes age related oxidative stress, damaged TM, increased IOP, and RGC death demands a multifunctional intervention. Sydnonimine class of NO donor such as linsidomine is in clinical use for many decades for the treatment of angina pectoris and atherosclerosis as its carbamate prodrug molsidomine [24]. Molsidomine has no NO release profile or IOP lowering activity in monkey eyes (private communication, unpublished result) and one of the possible reasons is lack of enzymatic hydrolysis of carbamate functional group of this prodrug to release free drug linsidomine in ocular tissues. The free drug linsidomine or SIN-1 is tested in rabbit eyes and known to reduce IOP at a very high dose (20 mM) via intraocular injection [25], and the effect lasted only up to 3 h post dose, indicating the NO release from linsidomine (SIN-1) is too fast, difficult to control, and may possibly deplete quickly in the presence of pathological superoxide radicals and convert to peroxynitrite radicals. We started the design and synthesis of **SA-9** with two goals; to scavenge broad spectrum ROS and improve NO bioavailability with optimized physicochemical property to allow delivery of the compound as an eye drop formulation with a better shelf-life.

We took advantage of sulfur’s ability to scavenge a broad spectrum of free radicals and designed and synthesized the hybrid compound **SA-9** containing both NO donating sydnonimine and antioxidant sulfide moieties. Sulfur is an important element in nature and part of all the important antioxidant amino acids and peptides including homocysteine, methionine, glutathione, taurine, and lipoic acids [26]. They participate in the redox reactions to scavenge hydroxyl (OH.), superoxide (O_2_.^−^), as well as hypochlorous radicals (HOCl). Fernandes et al. [27] reported that the anti-inflammatory activity of Sulindac, actually comes from its active metabolites; Sulindac sulfides and Sulindac sulfones after the parent prodrug Sulindac gets metabolized in vivo with enhanced radical scavenging activity against both ROS and RNS. Bogatyrenko et al. [28] have reported that, while a polyphenolic anti-oxidant sodium S-[3-(3-tert-butyl-4-hydroxyphenyl) propyl] thiosulfate (TC-13) has no antitumor activity itself, the physical combination of TC-13 with the nitric oxide donor (NaNO_2_) increases the antitumor activity in an experimental mice model. In the biological system, iron nitrosyls and nitrosothiols are the most relevant agents responsible for storage and transport of the NO and related compounds. Most of the target receptors of NO also contain iron centers and/or thiol groups. The most important exogenous sources of nitric oxide (NO-donors) are nitrosothiols and metal nitrosylcomplexes [29], but they have very short half-lives. We recognize the major challenge in designing hybrid molecules is that they should not provide the synchronous ROS scavenging and NO release activities because of the simple reason that, NO is also a free radical and can be scavenged by the antioxidant functionality present in the molecule itself if it gets released first. Here, in both buffer solution and TM cells, we found that all three compounds (**SA-2**, **SA-9**, and **SA-10**) can scavenge superoxide (O_2_^−^) radicals as early as 7 min where the NO release is delayed and started after 24 h. We believe this schedule will provide the molecule a good opportunity to scavenge the unwanted free radicals produced during glaucomatous pathology after first encounter and will protect the NO from being destroyed. This unique sequential action of **SA-2**, **SA-9**, and **SA-10** was anticipated to provide robust cellular protection, vasorelaxation, and longer duration of anticipated bioactivities. Note that, **SA-10** is the oxidative metabolite of **SA-9** and is more hydrophilic by nature with the propensity for faster clearance. Additionally, **SA-10** demonstrated similar potency and efficacy in scavenging ROS and releasing NO in cells, but the peroxynitrite generating activity was higher than the parent compound **SA-9**. Therefore, we selected **SA-9** to evaluate first in the animal model.

Our first-generation hybrid NO donor with SOD mimetic activity **SA-2** though was capable of scavenging superoxide and provide NO to ocular tissues, the highly hydrophilic nature was not suitable for topical ocular drug delivery. Here, we have achieved the successful delivery of **SA-9** to both anterior and posterior segments of the eye after eye drop instillation and quantified **SA-9** in ocular tissues in ng/mg ranges. We still need to continue this investigation and determine the full pharmacokinetic profile and dosing regimen to finalize an effective dose. However, what is a more interesting find is the nanosuspension formulation 2% **SA-9 NPs** that contained a nearly 700-fold less quantity of **SA-9** than the solution formulation of 2% free **SA-9** and when dosed topically still provided **SA-9** in ocular tissues in the pg/mg range and significantly lower IOP in mouse eyes. PLGA encapsulation has been reported to deliver small molecules via trans-corneal permeation to the eyes to improve intraocular bioavailability of the encapsulated drug [30,31,32]. Dorzolamide-loaded PLGA/vitamin E TPGS was tested as an eye drop to enhance corneal permeability and sustained drug action [31]. Tahara et al. [32] reported the feasibility of drug delivery to the eye’s posterior segment by topical instillation of PLGA nanoparticles. PLGA is an FDA approved biodegradable polymer with an excellent safety profile. Though we have not conducted a detailed toxicity study of this nanosuspension formulation in mouse eyes, we have not encountered any visual clinical sign or symptoms at this dose and duration of the study. Further toxicity assessment will continue. We agree mouse eyes do not represent human eyes and the volume and distribution of drug will vary, however, this preclinical OHT mouse model developed and validated by Patel et al. recapitulate the compromised TM due to ER stress and ROS elevation, extracellular matrix (ECM) remodeling, and decreased aqueous humor clearance, all resulting in increased ocular hypertension and providing a good platform to test our novel hybrid compounds. Further mechanism of action in both cell animal models will be continued.

## 5. Conclusions

In summary, we designed and synthesized a novel sulfur containing hybrid NO donor-antioxidant small molecule **SA-9** and its active oxidative metabolite **SA-10** with optimal drug like properties. Both compounds were synthesized using a three-step synthetic procedure and can be scaled up easily. The compounds demonstrated acceptable aqueous solubility and stability at biological pH necessary for solution formulation. Compounds **SA-9** and **SA-10** demonstrated immediate broad-spectrum ROS scavenging activities followed by delayed NO release profile in both buffer and TM cells while maintaining NO bioavailability. We provided evidence of **SA-9**-induced cytoprotection in TM cells and a slow-release profile achieved when **SA-9** was encapsulated as PLGA nanosuspension. Biodistribution study demonstrated sufficient quantity of **SA-9** in both anterior and posterior segment of mouse eyes after single eye drop instillation of either free drug **SA-9** or nanosuspension **SA-9 NPs**. Ultimately, **SA-9 NPs** significantly reduced IOP in dexamethasone induced OHT mouse eyes. Altogether, **SA-9** is an attractive experimental molecule with multifunctional biological activities useful for treatment of glaucoma and related optic neuropathy conditions associated with increased oxidative stress and decreased level of nitric oxide.

## Data Availability

Data is contained within the article or Supplementary Materials.

## Supplementary Materials

The following are available online at https://www.mdpi.com/article/10.3390/antiox10040575/s1, Figure S1: Superoxide scavenging ability of SA compounds in buffer and cells, Figure S2: Quantitative measurement of peroxynitrite radical formation after treatment of SA compounds in NTM-5 cells.
