# Supplementary material for: Novel Thiol Containing Hybrid Antioxidant-Nitric Oxide Donor Small Molecules for Treatment of Glaucoma
Source: Antioxidants (Basel). 2021 Apr 8;10(4):575. doi: 10.3390/antiox10040575 (PMC8068288; doi:10.3390/antiox10040575)
Supplement: Supplementary file 1 [file antioxidants-10-00575-s001.pdf]

antioxidants

MDPI

Article

Supplementary Material: Novel Thiol Containing Hybrid Antioxidant-Nitric Oxide Donor Small Molecules for Treatment of Glaucoma

Charles E Amankwa [1,2], Sudershan R Gondi [1,2], Adnan Dibas [1,2], Courtney Weston [1,2], Arlene Funk [1,2], Tam Nguyen [3], Kytai T Nguyen [3], Dorette Z Ellis [2,4] and Suchismita Acharya [1,2,4,*]

[1] Department of Pharmacology and Neuroscience, University of North Texas Health Science Center, Fort Worth, TX 76107, USA; CharlesAmankwa@my.unthsc.edu (C.E.A.); sudershan.gondi@unthsc.edu (S.R.G.); dibasa@yahoo.com (A.D.); courtney.weston@outlook.com (C.W.); Arlene.Funk@my.unthsc.edu (A.F.);
[2] North Texas Eye Research Institute, University of North Texas Health Science Center, Fort Worth, TX 76107, USA;
[3] Department of Bioengineering, University of Texas at Arlington, Arlington, TX 76010, USA; tam.nguyen12@mavs.uta.edu (T.N.); knguyen@uta.edu (K.T.N.)
[4] Department of Pharmaceutical Sciences, College of Pharmacy, University of North Texas Health Science Center, Fort Worth, TX 76107, USA, dorette.ellis@unthsc.edu
* Correspondence: suchismita.acharya@unthsc.edu

Citation: Amankwa, C.E.; Gondi, S.R.; Dibas, A.; Weston, C.; Funk, A.; Nguyen, T.; Nguyen, K.T.; Ellis, D.Z.; Acharya, S. Novel Thiol Containing Hybrid Antioxidant-Nitric Oxide Donor Small Molecules for Treatment of Glaucoma. *Antioxidants* **2021**, *10*, 575. https://doi.org/10.3390/antiox10040575

Academic Editor: Urara Hasegawa

Received: 28 February 2021
Accepted: 5 April 2021
Published: 8 April 2021

Publisher's Note: MDPI stays neutral with regard to jurisdictional claims in published maps and institutional affiliations.

Copyright: © 2021 by the authors. Licensee MDPI, Basel, Switzerland. This article is an open access article distributed under the terms and conditions of the Creative Commons Attribution (CC BY) license (http://creativecommons.org/licenses/by/4.0/).

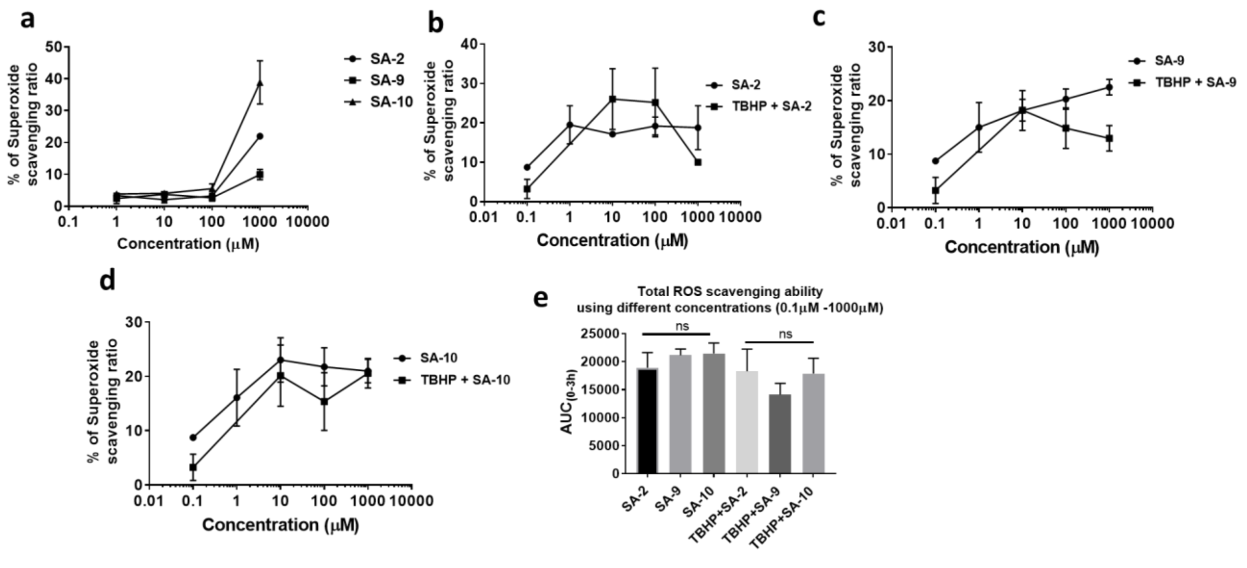

Figure S1: Superoxide scavenging ability of SA compounds in buffer and cells. **a)** The % of superoxide scavenging response from different concentrations of **SA-2**, **SA-9** and **SA-10** in pyrogallol (1 μM, 10 μM, 100 μM and 1,000 μM) induced superoxide induction assay at 15 minutes time point in buffer solution. The AUC_{0-15} for **SA-2, SA-9** and **SA-10** were 11604 ± 815.9, 5972 ± 834.9 and 20433 ± 3128 respectively. **b-d)** The superoxide scavenging ratio (%) after 18h treatment of different concentrations (0.1 μM, 1 μM, 10 μM, 100 μM and 1,000 μM) of **SA-2, SA-9** and **SA-10** to NTM-5 cell supernatant with or without TBHP (5.5 mM) using pyrogallol induced superoxide induction assay at 3h time point. Control is untreated cells. The cells were previously treated with 5.5 mM of TBHP for 30 minutes followed by SA compounds and TBHP and incubated for 18h. Control is untreated cells. **e)** The AUC_{0-3h} for **SA-2, SA-9** and **SA-10** were 18902 ±2714, 21155 ± 1099 and 21444 ± 1877 respectively. The AUC_{0-3h} for TBHP + **SA-2,** TBHP + **SA-9** and TBHP + **SA-10** were 18285 ±3960, 14120 ±2023 and 17893 ±2714 respectively. Three technical replicates were used for each experiment and all experiments were repeated 2 times. N = 3.

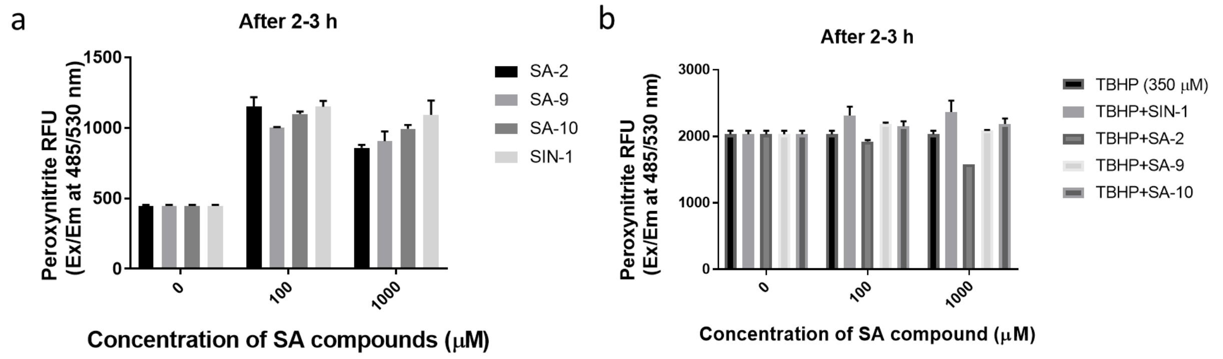

Figure S2: Quantitative measurement of peroxynitrite ($ONOO^{\cdot-}$) radical formation after treatment of SA compounds in NTM-5 cells. **a)** NTM-5 cells were labelled with peroxynitrite green sensor followed by treatment with 0.1 µM or 1 mM of SIN-1, **SA-2**, **SA-9** and **SA-10**. Changes in green fluorescence signal corresponded to level of $ONOO^{\cdot-}$ radicals as measured at Ex/Em of 485/530 nm after 2-3h. **b)** NTM-5 cells labelled with peroxynitrite green sensor followed by treatment with 0.1 µM or 1mM of SIN-1, **SA-2, SA-9** and **SA-10** with TBHP (350 µM). Changes in green fluorescence signal corresponded to level of $ONOO^{\cdot-}$ radicals as measured at Ex/Em of 485/530 nm after 2-3h. SIN-1 is used as positive control. N =3.
